# Learning by Demonstration for Motion Planning of Upper-Limb Exoskeletons

**DOI:** 10.3389/fnbot.2018.00005

**Published:** 2018-02-23

**Authors:** Clemente Lauretti, Francesca Cordella, Anna Lisa Ciancio, Emilio Trigili, Jose Maria Catalan, Francisco Javier Badesa, Simona Crea, Silvio Marcello Pagliara, Silvia Sterzi, Nicola Vitiello, Nicolas Garcia Aracil, Loredana Zollo

**Affiliations:** ^1^Research Unit of Biomedical Robotics and Biomicrosystems, Università Campus Bio-Medico, Rome, Italy; ^2^The BioRobotics Institute, Scuola Superiore Sant’Anna, Pisa, Italy; ^3^Biomedical Neuroengineering Research Group, Miguel Hernandez University, Elche, Spain; ^4^Departamento de Ingeniería en Automática, Electrónica, Arquitectura y Redes de Computadores, Universidad de Cádiz, Cádiz, Spain; ^5^GLIC—Italian Network of Assistive Technology Centers, Bologna, Italy; ^6^Unit of Physical and Rehabilitation Medicine, Università Campus Bio-Medico, Rome, Italy; ^7^Fondazione Don Carlo Gnocchi, Firenze, Italy

**Keywords:** motion planning, machine learning, learning by demonstration, dynamics movement primitives, assistive robotics

## Abstract

The reference joint position of upper-limb exoskeletons is typically obtained by means of Cartesian motion planners and inverse kinematics algorithms with the inverse Jacobian; this approach allows exploiting the available Degrees of Freedom (i.e. DoFs) of the robot kinematic chain to achieve the desired end-effector pose; however, if used to operate non-redundant exoskeletons, it does not ensure that anthropomorphic criteria are satisfied in the whole human-robot workspace. This paper proposes a motion planning system, based on Learning by Demonstration, for upper-limb exoskeletons that allow successfully assisting patients during Activities of Daily Living (ADLs) in unstructured environment, while ensuring that anthropomorphic criteria are satisfied in the whole human-robot workspace. The motion planning system combines Learning by Demonstration with the computation of Dynamic Motion Primitives and machine learning techniques to construct task- and patient-specific joint trajectories based on the learnt trajectories. System validation was carried out in simulation and in a real setting with a 4-DoF upper-limb exoskeleton, a 5-DoF wrist-hand exoskeleton and four patients with Limb Girdle Muscular Dystrophy. Validation was addressed to (i) compare the performance of the proposed motion planning with traditional methods; (ii) assess the generalization capabilities of the proposed method with respect to the environment variability. Three ADLs were chosen to validate the system: drinking, pouring and lifting a light sphere. The achieved results showed a 100% success rate in the task fulfillment, with a high level of generalization with respect to the environment variability. Moreover, an anthropomorphic configuration of the exoskeleton is always ensured.

## 1 Introduction

Understanding trajectory planning in human movements plays a paramount role in upper-limb exoskeletons for rehabilitation and assistive purposes because of the tight physical human-robot interaction. A typical strategy for determining the desired trajectory to be tracked by the exoskeleton in complex tasks, such as the Activities of Daily Living (ADLs), is to replicate human movements (An et al., 1988). Joint trajectories from unimpaired volunteers, caregivers, or therapists can be pre-recorded and later executed by the robotic system throughout specific mapping methods, i.e., spline decomposition (Jiang et al., 2013), or else optimization of *ad hoc* developed objective functions (Provenzale et al., 2014). However, these methods are successful in structured environments, since they cannot manage variability in the environment and external perturbations.

For ADLs in unstructured environment, a Cartesian motion planner can be conveniently adopted (Marchal-Crespo and Reinkensmeyer, 2009) and a purposely developed mathematical model of human motor behavior should be formulated in order to plan the desired trajectories in a way similar to humans. This is the case, for example, of the minimum jerk criterion (Flash and Hogan, 1985) or the minimum torque model (Svinin et al., 2010) for point-to-point reaching tasks.

For the exoskeletons, the approach based on Cartesian motion planning requires that inverse kinematics (IK) (Kim et al., 2012) is applied for computing joint motion, with the consequent increase of the computational burden. Moreover, the traditional IK algorithm with inverse Jacobian allows exploiting the available DoFs of the robot kinematic chain to achieve the desired end-effector pose; however, it does not guarantee that anthropomorphic criteria in the whole human-robot workspace are satisfied, especially in non-redundant structures. Alternative methods that account for anthropomorphic configurations in the joint space are based on the computation of the swivel angle. It can be estimated by means of geometric methods (Mihelj, 2006) or analytical methods based on the augmented Jacobian (Papaleo et al., 2015); however, in the case of non-redundant exoskeletons (as most of the commercially available ones (Marchal-Crespo and Reinkensmeyer, 2009)), the computation of the swivel angle causes the reduction of the number of Cartesian DoFs to be controlled, since the swivel angle is computed in lieu of one of the controlled Cartesian coordinates; as a consequence, this entails a reduction of the success rate in the fulfilment of the ADLs. Other approaches are based on hybrid Cartesian joint motion planners (Pattacini et al., 2010); nevertheless, as for the methods based on the computation of the swivel angle, they cannot be adopted in non-redundant exoskeletons without reducing the Cartesian DoFs to be controlled.

An alternative approach is represented by Learning By Demonstration (LbD), where the human subject is observed during the task execution and the robotic system replicates the learnt movement. It allows avoiding motion planning in the Cartesian space and inverse kinematics, but it requires to learn the target joint configuration to be reached through supervised learning. In literature, supervised learning methods, based on NNs, are widely used by researchers to learn the IK of redundant and non-redundant robots as in Oyama et al. (2001). Due to their adaptability to several contexts, NNs are employed in several robotic applications. In Li et al. (2017), they are used for redundancy resolution in presence of noise; in Jin and Li (2016) and Jin et al. (2017), NNs are adopted for motion control of multiple cooperating redundant manipulators; and in Noda et al. (2014), they are used for robot motion generation based on data from multimodal sensory systems. Nevertheless, to the best of our knowledge, how learning methods based on NNs can improve performance of LbD approaches during the learning of motion features and robot IK is not fairly explored.

This work proposes a motion planning system grounded on LbD for generating reference trajectories in the joint space for upper-limb exoskeletons, starting from the observation of the human motion during the execution of ADLs. The paper contribution is mainly addressed to extend the LbD approach in Ijspeert et al. (2013) for the control of upper-limb exoskeletons and to significantly improve it by introducing a Neural Network (NN), that learns the motion features and the robot inverse kinematics. The proposed method offers the following three main advantages with respect to the available techniques used in literature to plan the motion of upper-limb exoskeletons (i.e., motion planning in the Cartesian space and inverse kinematics): (i) it does not require the formulation of mathematical models of human motor behavior in order to accomplish the task in a way similar to humans; (ii) it allows performing the task also in unstructured environments (where a variability can be caused, for example, by the object position changes and subject different anthropometries); (iii) it guarantees the task accomplishment in the feasible workspace by preserving anthropomorphic configurations of the assisted human arm.

The proposed motion planner is based on Dynamic Movement Primitives (DMPs), with a well-defined landscape attractor (Ijspeert et al., 2013). This attractor allows replicating the recorded trajectory by means of a weighted sum of optimally spaced Gaussian Kernels; weight parameters (DMP parameters) are extracted from demonstrated movements with a Locally Weighted Regression (LWR) algorithm and are used to train a neural network through supervised learning. The neural network has the aim to define DMP parameters and joint target position and receives in input context factors (such as object position or task type). The DMP parameters are then processed by the DMP computation module that provides the exoskeleton reference joint trajectories.

The proposed motion planner was tested on an upper-limb exoskeleton during ADLs tasks. The exoskeleton was made of a 4-DoF shoulder-elbow exoskeleton (i.e., NeuroExos Shoulder-Elbow Module (NESM) (Crea et al., 2016)) for reaching movements, and a 5-DoF wrist-hand exoskeleton responsible for the grasping phase. The system was experimentally validated on four patients with Limb Girdle Muscular Dystrophy (LGMDs). They were asked to perform one ADL (i.e., the drinking task) and two activities belonging to the Southampton Hand Assessment Procedure (SHAP) clinical test (i.e., pouring and lifting a light sphere, consisting in reach-grasp-move-release a spherical object). The position of the object to be grasped was acquired by means of an external camera (Optitrack).

A comparative analysis with the traditional approach based on path planning and IK for upper-limb exoskeletons was carried out. Moreover, the data acquired during the experimental session were used to assess the generalization capability of the proposed motion planning system with respect to the different anthropometry of the patients and the different object positions. Performance of the proposed motion planning system was measured through a set of performance indicators, consisting of success rate, distance from target position, distance from the physiological behavior, and computational burden.

The paper is organized as follows: in Section 2, the exoskeleton, the proposed motion planner, and the experimental setup and protocol are presented. Experimental results are illustrated and discussed in Section 3 and Section 4, respectively. Finally, conclusion and future works are reported in Section 5.

## 2 Materials and Methods

### 2.1 Exoskeletons

**Figure 1:**
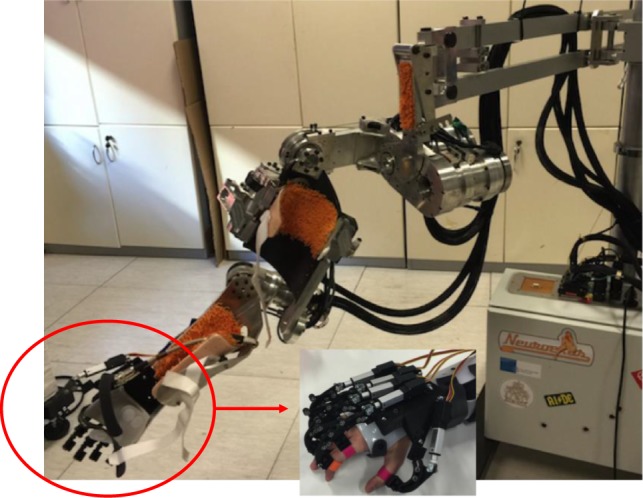
NESM upper-limb exoskeleton with the wrist-hand exoskeleton.

The upper-limb exoskeleton used to validate the proposed motion planning system is shown in Figure 1. It consists of the NESM 4-DoF exoskeleton and a 5-DoF Wrist-hand exoskeleton, described in the following.

#### 2.1.1 NESM

NESM is an upper-limb exoskeleton consisting of four active DoFs addressing the shoulder abduction/adduction (sA/A), flexion/extension (sF/E) and internal/external rotation (sI/E), as well as the elbow flexion/extension (eF/E) movements (Crea et al., 2016). Additional passive degrees of freedom and size regulations are included within the kinematic chain to improve the safety and wear ability of the device: this system automatically compensates for joint misalignments of the elbow and shoulder complex and allows users with different anthropometries wearing the device.

Each actuation unit has a series-elastic actuator (SEA), comprising a DC motor and reduction gear in series with a custom spring. Two absolute encoders placed at both sides of the spring allow sensing the joint torque by measuring the spring deformation, and at the same time, the encoder mounted more proximally to the human joint provides the joint angular value. By virtue of the SEA architecture, both position and torque control strategies have been implemented.

The sA/A and sF/E actuation units are identical and are able to withstand peak torques up to 60 *Nm*. Similarly, the sI/E and eF/E actuation units can deliver up to 30 *Nm* of peak torques. These features make the exoskeleton suitable to assist users having highly reduced or null residual motion capabilities of their upper arm. Notably, in this study, the position control modality is employed to perform completely passive mobilization of the user’s arm.

Each joint can move within the following range of motion (the zero configuration is with the arm parallel to the trunk): 0° to −90° for sA/A and sF/E, −75° to 25° for sI/E and 0° to 120° for eF/E.

#### 2.1.2 Wrist-Hand Exoskeleton

The wrist-hand exoskeleton is composed of two modules, the hand and the wrist, that can be used separately or in combination. The wrist exoskeleton guarantees the activation of the prono/supination movements. It consists of a DC motor with a reduction stage, which drives a geared ring guide. The guide is attached to an orthosis that aligns the forearm with the guide axis. Joint limits are mechanically provided, but, if necessary they can be reduced via software for increasing the safety in the human-robot interaction.

The hand exoskeleton has 4 active DoFs: F/E of the index finger Metacarpophalangeal (MCP) joint, F/E of the middle finger MCP joint, F/E of the ring and little finger MCP joints, and F/E of the thumb MCP joint. A linkage mechanism between the M regulator as well. When a reference MCP and the Proximalinterphalangeal (PIP) joint is adopted on each finger and is driven by a linear actuator, for moving both PIP and MCP joints. A unique linear actuator is used for driving the PIP and MCP joints of both the third and the fourth fingers. The thumb A/A is fixed in a suitable position.

The wrist exoskeleton can be easily connected to the shoulder-elbow exoskeleton. In fact, by simply removing the forearm cuff from the NESM, the cuff integrated to the wrist exoskeleton can be attached to the output frame of the elbow actuation unit. The resulting device is a full-arm robotic exoskeleton.

### 2.2 Low Level Control (LLC)

The control system used to operate the NESM implements two control strategies: joint position and joint torque control modes. When controlled in position, each actuation unit drives the joint position along a reference value or trajectory. The controller is based on a proportional-integral-derivative (PID) regulator, which operates on the difference between the reference joint angle and the measured one. The output is a current commanded to the driver of the SEA actuation unit to provide the torque necessary to achieve the movement with null steady-state error.

In the torque control mode, each motor is controlled to provide a certain amount of torque. The closed-loop torque controller output is dependent on the difference between the desired joint torque and the measured one, and it is built on a PID regulator as well. When a reference torque of 0 *Nm* is commanded on each joint, the device can be used in transparent mode, allowing the user to freely move the arm. Conversely, the wrist module and the hand exoskeleton could be controlled only in position; the controller used to operate these devices is based on a PID regulator, which operates on the difference between the reference joint angle and the measured one.

### 2.3 Motion Planning Based on LbD for Upper-Limb Exoskeletons

**Figure 2:**
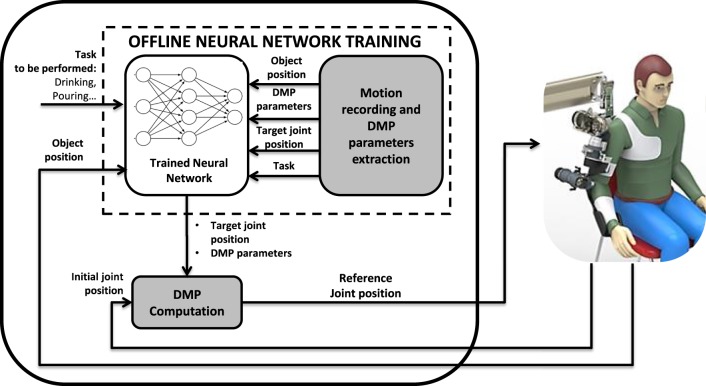
Block scheme of the proposed motion planning system.

The proposed motion planning for upper-limb exoskeletons is shown in Figure 2. A variation of LbD method used in Ijspeert et al. (2013) is presented. In particular, in this work, differently from Ijspeert et al. (2013), a combination of DMP and supervised learning is adopted with the aim of avoiding motion planning in the Cartesian space and inverse kinematics. The proposed motion planning consists of two main stages, named off-line neural network training and DMP computation. In the off-line neural network training, the trajectories executed by a healthy human subject, e.g., the therapist or the caregiver, are recorded by means of motion tracking devices such as magneto inertial sensors or the robot itself when backdriven, and distinctive features, named DMP parameters, are subsequently extracted using a LWR algorithm (“Motion recording and DMP parameters extraction” block in Figure 2). Hence, a neural network is trained through the Levenberg-Marquardt (LM) supervised learning algorithm in order to associate DMP parameters and robot joint target position to context factors taken in input (i.e., object position and task to be performed).

In the DMP computation, the patient can perform an ADL task with the assistance of the exoskeleton. Depending on the task and object position, the trained neural network provides the proper set of DMP parameters and robot joint target positions for computing the set of DMPs that best fit the desired task (“DMP computation” block).

#### 2.3.1 DMP Computation

The computation of the DMPs is obtained through the resolution of a non-linear second order system, expressed as

(1) $$\tau \ddot{q}=\alpha_{q}\left(\beta_{q}\left(g-q\right)-\dot{q}\right)+f$$

where *τ* is a time constant, *α_q_* and *β_q_* are positive constants, *q*_0_ and *g* are the initial and final points of the trajectory, respectively, and *f* is a forcing term that implements the landscape attractor of the system. In equation (1), *q* refers to a generic joint position of the robot that needs to be computed for each joint of the exoskeleton (i.e., *q*_1_, *q*_2_ … *q*_5_).

A possible formulation of the forcing term, namely the landscape attractor (Ijspeert et al., 2013), is

(2) $$f\left(x\right)=\frac{\sum_{i=1}^{N}\;\Psi_{i}\left(x\right)\omega_{i}}{\sum_{i=1}^{N}\;\Psi_{i}\left(x\right)}x\left(g-q_{0}\right)$$

where *ω_i_* is the DMP parameters, namely the weight parameters adopted to reconstruct the recorded motion, while *x* is the state variable of the system that makes equation (1) a time-independent system. It is defined as

(3) $$\tau ẋ=-\alpha_{x}x$$

where *α_x_* is a positive constant. On the other hand, Ψ*_i_*(*x*) is Gaussian kernels expressed as

(4) $$\Psi_{i}\left(x\right)=\exp\left(-\frac{1}{2\sigma_{i}^{2}}{\left(x-c_{i}\right)}^{2}\right)$$

where *σ_i_*, *c_i_*, and *N* are the width, the centers, and the number of Gaussian functions, respectively. The state variable *x* as well as centers *c_i_* range between 0 and 1.

**Figure 3:**
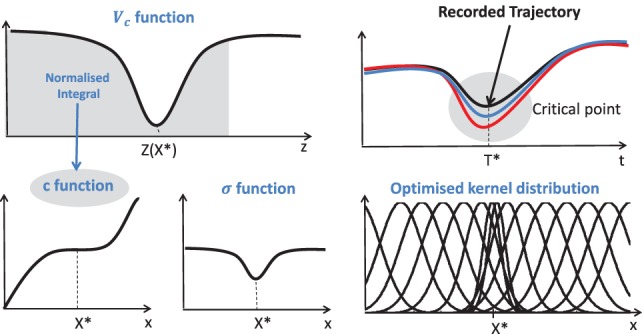
*c* and *σ* functions for the optimal allocation of the Gaussian Kernels. X* and T* are the state value and time instant corresponding to the critical point (Lauretti et al., 2017a).

As in our previous work (Lauretti et al., 2017a), an optimized spatial allocation of the Gaussian kernels is adopted, depending on the complexity of the recorded trajectory. Hence, *c_i_* and *σ_i_* are defined as

(5) $$c\left(x\right)=\frac{\int_{0}^{x}\;V_{c}\left(z\right)\;\mathrm{dz}}{||\int_{0}^{1}\;V_{c}\left(z\right)\;\mathrm{dz}||}$$

(6) $$V_{c}\left(z\right)=1-\alpha_{z}\overset{P}{\underset{k=1}{\sum }}\;\exp\;\left(-\beta_{z}\left(z-z_{k}\right)\right)$$

(7) $$\sigma \left(x\right)=\gamma_{z}\frac{V_{c}\left(x\right)}{N}+\delta_{z}$$

(8) $$c_{i}=c\left(\frac{i}{N}\right)$$

(9) $$\sigma_{i}=\sigma \left(\frac{i}{N}\right)$$

where *α_z_*, *β_z_*, *γ_z_*, and *δ_z_* are positive constants, *P* is the number of critical points of the recorded trajectory, and *z_k_* is the normalized time instant of each critical point. A graphical representation of *c* and *σ* functions is provided in Figure 3.

#### 2.3.2 Off-Line Neural Network Training

##### 2.3.2.1 DMP Parameters Extraction

DMP parameters *ω_i_* are extracted through a LWR algorithm (Schaal and Atkeson, 1998). The recorded motion and derivatives, i.e., *q_d_*, ${\dot{q}}_{d}$, and ${\ddot{q}}_{d}$ are inserted in the forcing term in equation (2) as follows

(10) $$f_{t}=\tau {\ddot{q}}_{d}-\alpha_{q}\left(\beta_{q}\left(g-q_{d}\right)-{\dot{q}}_{d}\right),$$

and a function approximation problem is formulated. Hence, a locally weighted quadratic error is minimized by means of the following cost function

(11) $$J_{i}=\overset{P}{\underset{t=1}{\sum }}\;\Psi_{i}\left(t\right){\left(f_{t}\left(t\right)-\omega_{i}\epsilon \left(t\right)\right)}^{2}$$

(12) $$\epsilon \left(t\right)=x\left(g-q_{0}\right)$$

and *ω_i_* parameters that make *f_t_* as close as possible to *f* are found, for each kernel function Ψ_i_(*t*), in order to reconstruct the trajectory *q_d_*, ${\dot{q}}_{}$, and ${\ddot{q}}_{d}$, through *q*, $\dot{q}$, and $\ddot{q}$, respectively. In equation (12), ϵ is the error between the target joint position to be reached *g* and the initial joint position of the exoskeleton *q*_0_.

##### 2.3.2.2 Neural Network

A Levenberg-Marquardt algorithm (LM) has been adopted for the off-line neural network training (Lourakis, 2005). Given a parameter vector $p\in R^{n}$ and a measurement vector ***x*** ∈ ℜ*^m^*, the LM algorithm finds the functional relation (*f*) that maps the parameter vector ***p*** into an estimated measurement ***x***^ ($\hat{\text{x}}=f\left(\text{p}\right)$). A linear approximation of *f* in the neighborhood of ***p*** is provided by a Taylor series expansion

(13) $$f\left(\text{p}+\delta_{\text{p}}\right)=f\left(p\right)+\text{J}\delta_{p}+o\left(\text{p}\right)$$

Neglecting the higher order terms *o*(***p***), equation (12) could be approximated as

(14) $$f\left(\text{p}+\delta_{p}\right)\approx f\left(\text{p}\right)+\text{J}\delta_{p}$$

where ***J*** is the Jacobian matrix $\frac{\delta f\left(\text{P}\right)}{\delta \text{P}}$.

At each step of the iterative process, LM looks for the *δ_p_* that minimizes the error defined as $∥x-f\left(\text{p}+\delta_{p}\right)∥=∥x-f\left(\text{p}\right)+\text{J}\delta_{p}∥\;=∥\epsilon -\text{J}\delta_{p}∥$. The error is minimized when ***J****δ_p_*–ϵ is orthogonal to the column space of ***J***, namely when the following condition holds

(15) $${\text{J}}^{T}\left(\text{J}\delta_{p}-\epsilon_{p}\right)=0$$

(16) $${\text{J}}^{T}\text{J}\delta_{p}={\text{J}}^{T}\epsilon_{p}.$$

In the LM method, equation (16), called *normal equation*, is written as

(17) $$\text{N}\delta_{p}={\text{J}}^{T}\epsilon_{p}$$

(18) $$\text{N}=\mu +{\text{J}}^{T}\text{J}$$

where ***J****^T^****J*** and *μ* are called damping and damping term, respectively. One iteration of LM algorithm consists of finding an acceptable value of the damping term that reduces the error *ϵ_p_*. In other words, if *δ_p_* computed from equation (17), leads to a reduction of the error *ϵ_p_*, the damping term is decreased and the following iteration is processed; otherwise, the damping term is increased and equation (17) is solved again. The LM algorithm stops running when, at least, (i) ${\text{J}}^{T}\epsilon_{p}$ of equation (17) is lower than a preset threshold ϵ_1_ or (ii) *δ_p_* is lower than a threshold ϵ_2_ or (iii) a maximum number of iteration *N_MAX_* is reached. For the sake of brevity, the complete LM algorithm is not shown; further theoretical details about the implemented method could be found in Lourakis (2005).

**Figure 4:**
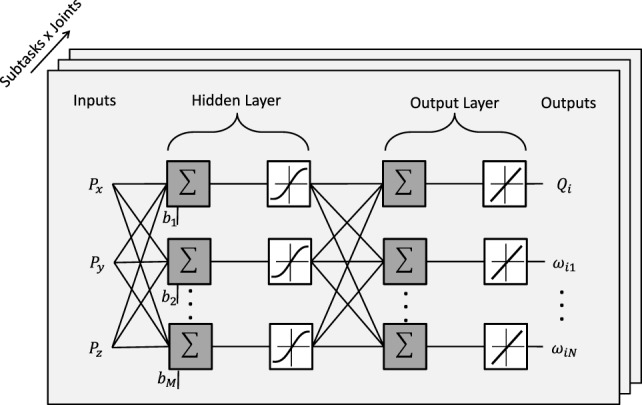
Structure of the adopted neural network.

The structure of the adopted neural network is reported in Figure 4. A two layer feed-forward network with *M* sigmoid hidden neurons and *N* + 1 linear output neurons is used for each joint and for each task the user wants to perform. The inputs of each network are the Cartesian target positions to be reached, *P_x_*, *P_y_*, and *P_z_* (e.g., object position); on the other hand, the outputs of each network are the DMP parameters, *ω*_1_, *ω*_2_…*ω_N_*, and the target joint angles, *Q_i_* (N is the number of DMP parameters computed for the i-th joint).

##### 2.3.2.3 Adapting NN Outputs to Different Subject Anthropometries

**Figure 5:**
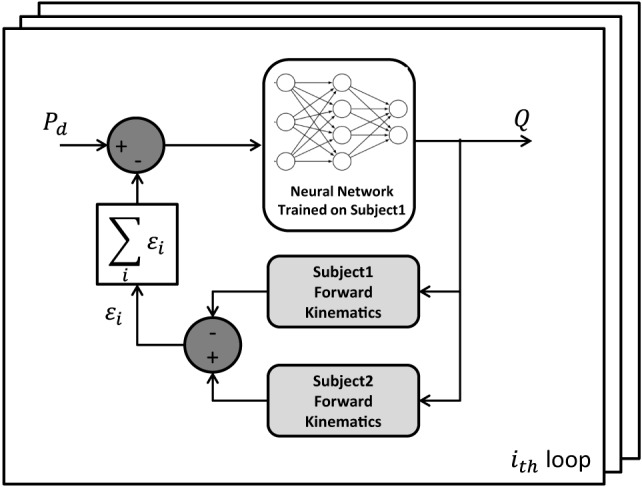
Block scheme of the recursive method used to adjust the NN outputs for different subject anthropometries.

In order to adapt the proposed method to different human bodies, a recursive method that adjusts the NN outputs for distinct subject anthropometries was used. Its functioning principle is shown in Figure 5.

In the block scheme, *P_d_* is the Cartesian target position to be reached, *Q* is the output configuration of the robot joints, subject 1 is the person involved in the NN training phase while subject 2 is the assisted person, who wants to perform an ADL thanks to the exoskeleton assistance. It is worth noting that, with the aforementioned exoskeletons and the described tasks, two loops of the recursive algorithm are suitable to obtain an acceptable error in reaching the target position (less than 10 mm).

### 2.4 Traditional Path Planning and IK

A simple path planning, based on a third-order polynomial function, was implemented in order to generate Cartesian trajectories with null velocity at the beginning and at the end of the movement. It can be written as

(19) $$z=-2\frac{z_{f}-z_{i}}{D^{3}}t^{3}+3\frac{z_{f}-z_{i}}{D^{2}}t^{2}+z_{i}$$

where *z* is the desired exoskeleton Cartesian pose, z*_f_* and *z_i_* are the final and initial desired Cartesian pose, respectively, and *D* is the motion duration.

Hence, two IK methods were adopted to generate the reference joint position for the exoskeleton. They are IK based on the computation of the swivel angle (named in the following *IK with swivel angle*) and IK with the inverse Jacobian (named in the following *IK Inverse Jacobian*).

#### 2.4.1 IK with Swivel Angle

**Figure 6:**
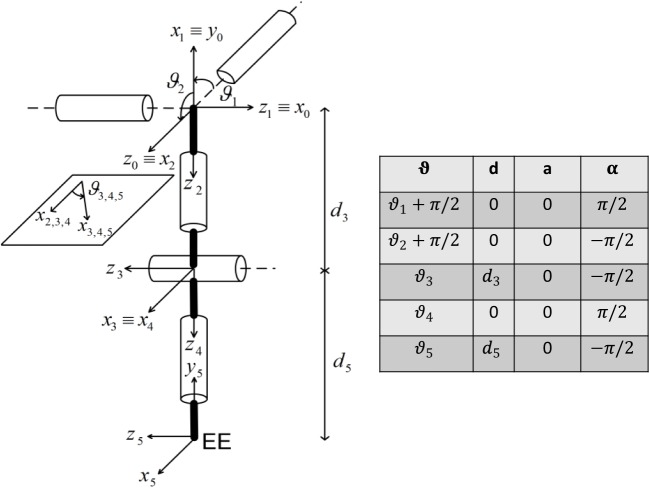
NESM reference frames positioning according to the Denavit–Hartenberg (D–H) convention.

The IK algorithm with swivel angle was *ad hoc* developed for a 4-DoF spherical-revolute (S-R) manipulator (i.e., the shoulder-elbow exoskeleton), based on geometrical considerations. An additional constraint was imposed to calculate the analytical solution for the last revolute DoF of the upper-limb exoskeleton (i.e., the wrist prono-supination). For the sake of clarity, the Denavit-Hartenberg model and parameters of the upper-limb exoskeleton are reported in Figure 6.

The IK algorithm for the shoulder-elbow exoskeleton manages three Cartesian coordinates and one orientation coordinate and consists of the following steps:

- Being the target position known (vector $\vec{p}$), the solution for the elbow angle is derived geometrically:
  (20) $$q_{4}=\pi -\mathrm{acos}\;\left(\frac{d_{3}^{2}+d_{5}^{2}-{\left|\vec{p}\right|}^{2}}{2d_{3}d_{5}}\right)$$
- The orientation coordinate is a free parameter, (*γ*), introduced for guaranteeing anthropomorphic criteria and is defined as the angle, on the frontal plane (*x*_0_–*y*_0_ in Figure 6), between the plane containing the upper arm and forearm and the frontal plane. Once *γ* is chosen, two possible configurations of the elbow (i.e., left or right) allow the arm lying in the chosen plane: the solution with the four angles in the physiological range is selected.
- Then, the shoulder joint angles can be derived from forward kinematics:
  (21) $$q_{1}=\mathrm{atan}\left(\frac{y_{o_{3}}}{x_{o_{3}}}\right)$$
  (22) $$q_{2}=\mathrm{acos}\left(\frac{z_{o_{3}}}{d_{3}}\right)$$
  (23) $$q_{3}=-\text{arccos}\left(\frac{z_{\mathrm{ee}}-d_{3}\;\text{cos}\;q_{2}-d_{5}\;\text{cos}\;q_{2}\;\text{cos}\;q_{4}}{d_{5}\;\text{sin}\;q_{4}\;\text{sin}\;q_{2}}\right)$$
- The wrist prono-supination angle is calculated, by imposing a constraint on the hand orientation. For the addressed tasks (i.e., drinking, pouring, reaching-grasping-moving-releasing of the sphere), two configurations were considered:
  (1) Palm of the hand pointing downward (for pouring and sphere reaching-moving):
    (24) $$q_{5}=\text{arctan}\left(\frac{\text{cos}\;q_{4}\left(\text{cos}\;q_{1}\;\text{sin}\;q_{3}+\;\text{cos}\;q_{2}\;\text{cos}\;q_{3}\;\text{sin}\;q_{1}\right)+\;\text{sin}\;q_{1}\;\text{sin}\;q_{2}\;\text{sin}\;q_{4}}{\text{cos}\;q_{2}\;\text{sin}\;\vartheta_{1}\;\text{sin}\;q_{3}-\text{cos}\;q_{1}\;\text{cos}\;q_{3}}\right)$$
  (2) Palm of the hand pointing left (for drinking):
    (25) $$q_{5}=\text{arctan}\left(\frac{\text{cos}\;q_{1}\;\text{cos}\;q_{3}-\text{cos}\;q_{2}\;\text{sin}\;q_{1}\;\text{sin}\;q_{3}}{\text{cos}\;q_{4}\left(\text{cos}\;q_{1}\;\text{sin}\;q_{3}\;+\;\text{cos}\;q_{2}\;\text{cos}\;qa_{3}\;\text{sin}\;q_{1}\right)+\text{sin}\;q_{1}\;\text{sin}\;q_{2}\;\text{sin}\;q_{4}}\right)$$

#### 2.4.2 IK with Inverse Jacobian

The IK algorithm with inverse Jacobian is well-described by the following equation (Siciliano et al., 2010),

(26) $$\dot{q}=J_{A}^{-1}\left(q\right)\left({ẋ}_{d}+\mathrm{Ke}\right)$$

where $J_{A}^{-1}$ is the analytical inverse Jacobian computed on the kinematic chain of Figure 6, *q* and $\dot{q}$ are the joint angle and its derivative, respectively, ${ẋ}_{d}$ is the desired velocity in the Cartesian space, K is a positive definite matrix (usually diagonal), and *e* is the *operational space error* defined as *e* = *x_d_*–*x_e_*. The desired joint configuration *q* is obtained by numerically integrating equation (26) through the Euler method.

### 2.5 Experimental Setup

**Figure 7:**
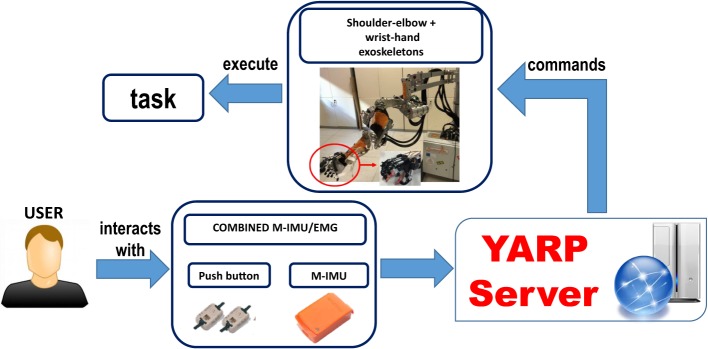
Block scheme of the platform.

The experimental platform for the validation of the proposed motion planning system based on LbD is shown in Figure 7.

A user graphical interface is used to show the action to perform to the subject. The control system architecture consisted in a finite-state machine, which divides the main task (i.e., drinking, pouring and reach-grasp-move-release a sphere) into several elementary actions (corresponding to the subtasks listed in Table 1) that the different devices can accomplish (e.g., waiting for the trigger, reaching the glass, grasping, etc.). Each subtask is triggered by the user by means of the combined M-IMU/EMG interfaces, letting him/her to control the exoskeletons. An abort function was also implemented in the state machine to safely abort the execution of the task at any time.

**Table 1:** Tasks description.

Task 1: Drinking	
subtask 1-1	reach the glass
subtask 1-2	reach the mouth
subtask 1-3	reach the table for releasing the glass
subtask 1-4	go back to the rest position

**Task 2: Pouring**	

subtask 2-1	reach the bottle
subtask 2-2	pour the water into the glass
subtask 2-3	reach the table for releasing the bottle
subtask 2-4	go back to the rest position

**Task 3: SHAP sphere**	

subtask 3-1	reach the sphere
subtask 3-2	move the sphere to another position on the table
subtask 3-3	go back to the rest position

The communication within the subsystems composing the platform is managed by the Yet Another Robotic Platform (YARP) messaging system. The motion commands acquired by the user are sent, through the YARP server, to the exoskeletons. All the acquired data are synchronized and saved under YARP.

**Figure 8:**
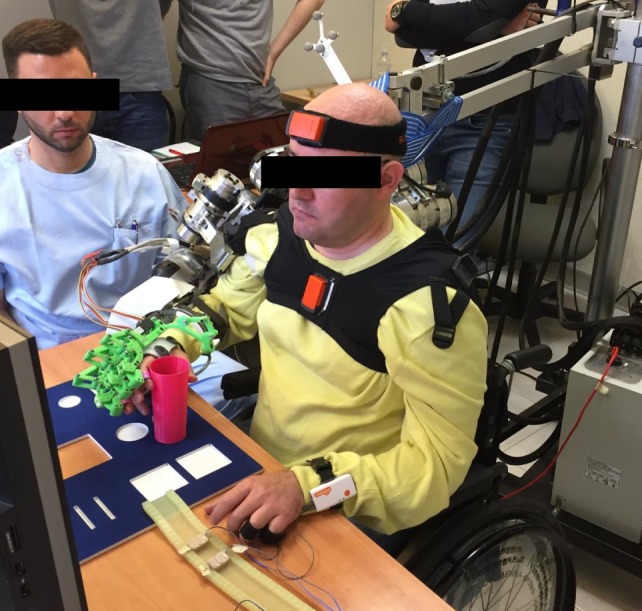
A representative subject performing the task (the subject signed an informed consent document to authorize publication of this picture).

The platform components are shown in Figure 8 and are detailed in the following.

#### 2.5.1 User Interface

The interface adopted to detect the user movement intention is based on the combined use of 2 push-buttons and 2 M-IMUs (Lauretti et al., 2017b).

The 2 push-buttons were placed on a table in order to be activated by the index and the thumb fingers and to be used as a switch. Moreover, the two M-IMUs (XSens MTw) were placed on the user trunk and head in order to detect his/her neck motion. An Awinda Station was used to record at 100 Hz of synchronized wireless data from the two M-IMUs.

By means of the developed interface the user may exploit: (i) the head yaw motion in the negative direction to operate the upper-limb exoskeleton movements and the head yaw motion in the positive direction to abort the task; (ii) the index finger and thumb residual motion to trigger the hand opening and the hand closing.

### 2.6 Experimental Protocol

#### 2.6.1 Off-Line Neural Network Training

**Figure 9:**
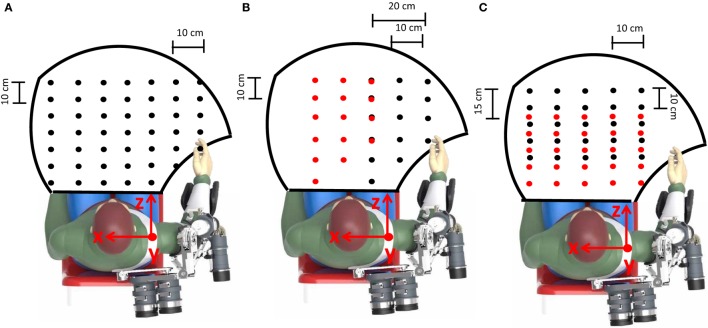
The workspace reached during the assistive tasks is delimited by the black line. Object positions during training are indicated by black dots [the glass in the drinking task in **(A)**, the bottle in the pouring task in **(B)** and the initial position of the sphere in the SHAP task in **(C)**]. Conversely, the glass positions during the pouring task and the sphere final positions in the SHAP task are indicated by red dots in **(B, C)**, respectively.

The developed neural network was trained off-line on a healthy volunteer subject (with upper arm length *L_Upper Arm_* = 0.33 *m* and forearm length *L_forearm_* = 0.3 *m*). He was asked to perform the drinking task, with 41 different glass positions (Figure 9A) and two activities belonging to the SHAP clinical test, i.e., pouring (for 15 different positions of the glass and the bottle as in Figure 9B) and reach-grasp-move-release a sphere (for 25 different positions of the sphere as in Figure 9C). Each task was performed 5 times per each object position and arm motion was recorded. The shoulder motion, i.e., the sA/A, sF/E, sI/E, and eF/E movements were recorded through the NESM used in transparent mode; conversely, the wrist Prono-Supination wP/S was recorded by means of two M-IMUs placed on the subject forearm and hand.

About 70% of the recorded data was used to train the neural network; the remaining 30% was used to validate and test the neural network in order to avoid over-fitting issues.

#### 2.6.2 DMP Computation and Control

The experimental session was aimed to measure performance of the proposed motion planning system, compare with the traditional approach based on inverse kinematics described in Section 2.4 and assess generalization capability. The system was tested during the fulfillment of the same ADLs used for training, i.e., drinking, the pouring, and reach-grasp-move-release a sphere. In the following, they are named task 1, 2, and 3, respectively. Additionally, each task is divided into a number of subtasks listed in Table 1.

The validation was performed in simulation and in the real setting with patients. Simulation tests allowed evaluating system performance in the whole human-robot workspace (238, 75, and 125 object positions were considered for task 1, 2, and 3, respectively). On the other hand, in the real setting, system performance was assessed on four patients with Limb Girdle Muscular Dystrophy (LGMDs). They, aged 38.5 on average (Standard Deviation 14.6), volunteered to participate in this study. The experimental protocol was approved by the local Ethical committee (Comitato Etico Università Campus Biomedico di Roma, reference number: 01/17 PAR ComEt CBM), by the Italian Ministry of Health (Registro—classif. DGDMF/I.5.i.m.2/2016/1096) and complied with the Declaration of Helsinki. The subjects were asked to perform three repetitions of the three tasks thanks to the assistance of the 4-DoF upper-limb and 5-DoF wrist-hand exoskeletons (3 object positions for each task were considered in this case).

##### 2.6.2.1 Comparative Analysis (CA) with Inverse Kinematics Methods

The CA was aimed to measure performance of the proposed motion planning based on LbD during the control of the exoskeleton and compare the results with the traditional approaches based on path planning and inverse kinematics described in Section 2.4. The comparative analysis (CA) was carried out in simulation on a subject modeled with 30 cm upper arm and forearm lengths and with 238, 75, and 125 different object positions.

##### 2.6.2.2 Generalization Capability Assessment (GCA)

The GCA was aimed to evaluate the generalization level of the proposed motion planning with respect to the different anthropometries of the patients and the different object positions. First, it was tested in simulation environment (GCA–sim) for 238, 75, and 125 object positions (for task 1, 2, and 3 respectively) per 25 different subject anthropometries, i.e., the combination of the following upper arm and forearm lengths: *L_Upper Arm_* = 30 cm, 32 cm, 34 cm, 36 cm, 38 cm and *L_forearm_* = 30 cm, 32 cm, 34 cm, 36 cm, 38 cm. Subsequently, the proposed motion planning was tested on the four patients (GCA–real), with *L_Upper Arm_* = 33 *cm* and *L_forearm_* = 30 cm, 30 cm, 35 cm, 37 cm, for 3 object positions per task.

System performance was measured through three quantitative indicators reported below.

##### 2.6.2.3 Performance Indices

The proposed performance indicators are: (i) *Position Err1*, *Orientation Err1*, *Position Err2*, *Orientation Err2*, (ii) PhJL (iii) Success Rate and (iv) mean cycle time. They are aimed at evaluating (i) distance from target position, (ii) distance from anthropomorphic configurations, taking into account the physiological joint limits, (iii) the success rate of the task execution, and (iv) the computational burden.

##### 2.6.2.4 Distance from Target Position

**Figure 10:**
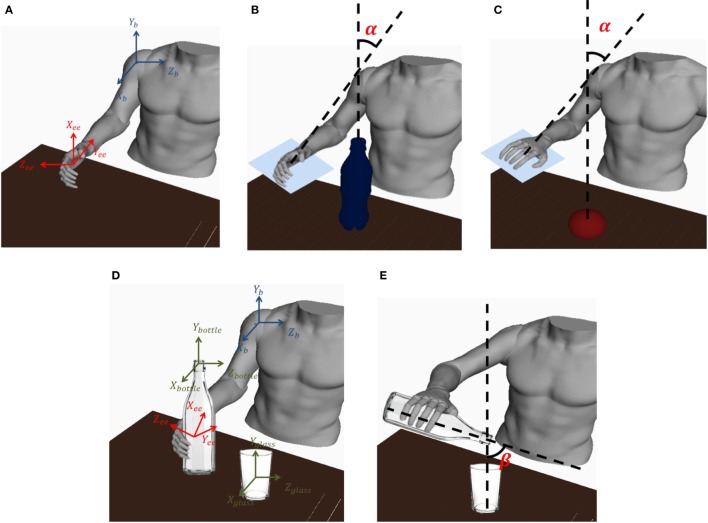
**(A)** a graphical representation of the end-effector and the base reference frame is shown; **(B)** the *α* angle for task 1-1, 2-1 is shown; **(C)** the *α* angle for task 3 is shown; **(D)** The base reference frame and bottle, end-effector, and glass reference frames are shown; **(E)** the *β* angle for task 2-2 is shown.

The error was measured during subtasks 1-1, 2-1, 3-1, and 3-2 (Table 1) as

(27) $$\mathrm{Position Err}=\frac{1}{2}\sqrt{{\left(x_{t}-x\right)}^{2}+{\left(y_{t}-y\right)}^{2}+{\left(z_{t}-z\right)}^{2}}$$

(28) $$\mathrm{Orientation Err}=∥\alpha_{t}-\alpha ∥$$

where *x_t_*, *y_t_*, and *z_t_* are the coordinates of the target position and *x*, *y*, and *z* are the coordinates of the actual position reached by the robot end-effector; *α_t_* is the desired angle *α* that needs to be 0 for a successful task fulfillment; *α* is illustrated in Figure 10 and is defined for subtask 1-1 and 2-1 as

(29) $$\alpha_{1}=\mathrm{acos}\left(\frac{X_{\mathrm{ee}}^{T}Y_{0}}{∥X_{\mathrm{ee}}∥\;∥Y_{0}∥}\right)$$

Conversely, for subtask 3-1 and 3-2 *α* is defined as

(30) $$\alpha_{2}=\mathrm{acos}\left(\frac{Z_{\mathrm{ee}}^{T}Y_{0}}{∥Z_{\mathrm{ee}}∥\;∥Y_{0}∥}\right)$$

where *Y*_0_, *X_ee_*, and *Z_ee_* are defined in the base reference frame [*X_B_*, *Y_B_*, *Z_B_*] as *Y*_0_ = [0, 1, 0], $X_{\mathrm{ee}}=T_{B}^{\mathrm{ee}}\left[1,0,0,1\right]$ and $Z_{\mathrm{ee}}=T_{B}^{\mathrm{ee}}\left[0,0,1,1\right]$ ($T_{B}^{\mathrm{ee}}$ is the base/end-effector transformation matrix).

For subtask 2-2, the position and orientation error are expressed as

(31) $${\mathrm{Position Err}}_{2-2}=\frac{1}{2}\sqrt{{\left(x_{\mathrm{bottle}}-x_{\mathrm{glass}}\right)}^{2}+{\left(z_{\mathrm{bottle}}-z_{\mathrm{glass}}\right)}^{2}}$$

(32) $${\mathrm{Orientation Err}}_{2-2}=∥\beta_{t}-\beta ∥$$

(33) $$\left[\begin{array}{c} x_{\mathrm{bottle}}\\ y_{\mathrm{bottle}}\\ z_{\mathrm{bottle}}\\ 1 \end{array}\right]=T_{B}^{\mathrm{ee}}\;T_{\mathrm{ee}}^{\mathrm{bottle}}\left[\begin{array}{c} 0\\ 0\\ 0\\ 1 \end{array}\right]$$

(34) $$\beta =\frac{\pi }{2}-\mathrm{acos}\left(\frac{X_{\mathrm{ee}}^{T}Y_{0}}{∥X_{\mathrm{ee}}∥\;∥Y_{0}∥}\right)$$

where *x_bottle_*, *z_bottle_*, *x_glass_*, and *z_glass_* are expressed in the [*X_B_*, *Y_B_*, *Z_B_*] reference frame (Figure 10), $T_{\mathrm{ee}}^{\mathrm{bottle}}$ is the end-effector/bottle-tip transformation matrix during the whole subtask 2-2 (i.e., when the hand exoskeleton is grasping the bottle) and *β*_t_ is the desired *β* that needs to range from 0 to $\frac{\pi }{3}$ in order to successfully accomplish the pouring task. Thus, defining *β_t_* = 0, an acceptable value of the orientation error, for a successful task fulfillment, ranges from 0 to $\frac{\pi }{3}$.

##### 2.6.2.5 Distance from the Physiological Joint Limits

The distance from the physiological joint limits is measured to assess the level of anthropomorphism of the reached configuration during motion. It is expressed as

(35) $$\mathrm{PhJL}=∥\frac{2\left(q_{i}-{\bar{q}}_{i}\right)}{q_{\mathrm{iM}}-q_{\mathrm{im}}}∥$$

where *q_i_* is the actual position of the i-th joint, *q_iM_* and *q_im_* are the upper and lower physiological limit of the i-th joint, respectively, and ${\bar{q}}_{i}$ is the mean value between *q_iM_* and *q_im_*. An acceptable value of *PhJL* for the considered tasks ranges in between 0 and 1.

##### 2.6.2.6 Success Rate of the Task Execution

The success rate of the task execution is evaluated as

(36) $$\mathrm{Success rate}=\frac{N_{\mathrm{succ}}}{N_{\mathrm{tot}}}\cdot 100$$

where *N_succ_* is the number of trials successfully accomplished and *N_tot_* is number of all the performed trials. Trials of tasks 1 and 3 are considered successfully accomplished if all the following conditions are satisfied:

- *Position Err* ≤ 15 mm,
- *Orientation Err* $\leq \frac{\pi }{12}$ rad,
- 0 ≤ *PhJL* ≤ 1.

Conversely, trials of Task 2 are considered successfully accomplished if all the following conditions are satisfied:

- *Position Err*_2−1_ ≤ 15 mm,
- *Orientation Err*_2−1_ $\leq \frac{\pi }{12}$ rad,
- *Position Err*_2−2_ ≤ 30 mm (i.e. the glass radius),
- 0 ≤ *Orientation Err*_2−2_ $\leq \frac{\pi }{3}\mathrm{rad}$,
- 0 ≤ *PhJL* ≤ 1.

The aforementioned ranges were experimentally retrieved.

##### 2.6.2.7 Computational Burden

The computational burden of the three compared methods is evaluated through the mean cycle time; it is the time required to complete one cycle of the algorithm that computes the desired joint trajectory starting from the object position and the task type. The computational time of the 3 methods was evaluated under the same hardware conditions (Processor: Intel(R) Core(TM)2 Duo CPU 3.00 GHz) and development environment (MATLAB R2014b).

##### 2.6.2.8 Statistical Analysis

For motion planner comparative analysis, mean value and SD of the computed performance indices were calculated for each task on the different object positions and subject anthropometries. For the generalization tests, mean value and SD of the computed performance indices were also calculated for all the subjects and the number of repetitions of each task. A statistical analysis based on Wilcoxon paired-sample test was performed for the comparative analysis between the proposed motion planning system and the traditional motion planner based on inverse kinematics. The analysis was carried out on multiple comparisons with Bonferroni correction; hence, significance was achieved for *p-value* < 0.05/*n_c_*, where *n_c_* is the number of multiple comparisons.

## 3 Results

**Figure 11:**
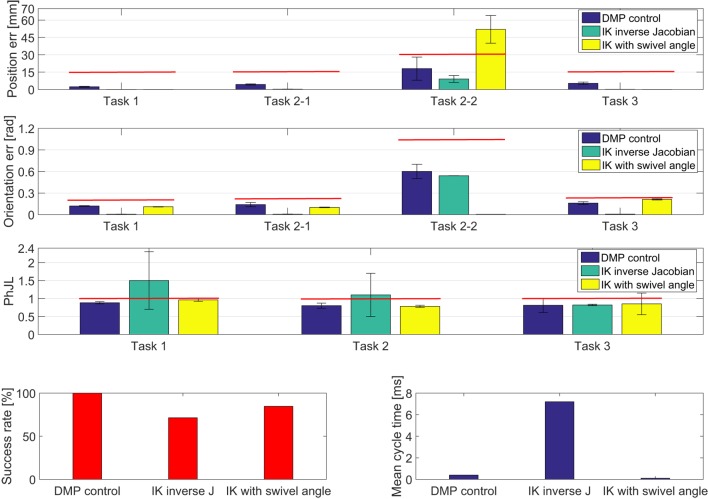
Experimental results obtained for CA. The red lines denote the range within which the task is considered successfully accomplished.

The results of the comparative analysis are reported in Figure 11. In particular, mean value and standard deviation of the position error, orientation error, and PhJL computed on the 238, 75, and 125 object positions (for task 1, 2, and 3, respectively) are reported.

One can observe that the DMP-based control always exceeded the other two algorithms based on inverse kinematics in terms of success rate. The DMP-based control always achieved 100% while the IK inverse Jacobian reached 71.4% and the IK swivel angle reached 84.7%. The differences are statistically significant with *p-value* < 0.0083 (for the DMP-based control compared to IK inverse Jacobian *p-value* = 0.0031 and for DMP-based control compared to IK swivel angle *p-value* = 0.0045).

On the other hand, as expected, the DMP-based control suffers from a position error higher than the one achieved with the other two algorithms (this difference is statistically significant with *p-value* = 0.0012 for the DMP-based control compared to IK inverse Jacobian and *p-value* = 0.0008 for DMP-based control compared to IK swivel angle), for all the subtasks except for subtask 2-2.

Indeed, about the position error of the subtask 2-2, the results achieved with the DMP-based control are comparable to the one achieved with IK inverse Jacobian (*p-value* = 0.09), but are better than the one achieved with IK swivel angle (*p-value* = 0.0033).

Conversely, the orientation error achieved for each task with the DMP-based control is comparable to the one achieved with IK with swivel angle (*p-value* = 0.12). The difference is statistically significant between the orientation error achieved by the DMP-based control and the one achieved with IK inverse Jacobian, which is lower (*p-value* = 0.0028).

Moreover, the results clearly show that the DMP-based control and IK with swivel angle ensure a more anthropomorphic configuration than IK inverse Jacobian, measured through *PhJL*. The differences are statistically significant, with *p-value* = 0.0024 for the comparison between DMP-based control and IK inverse Jacobian and *p-value* = 0.0019 for the comparison between IK swivel angle and IK inverse Jacobian.

Finally, considerations about the computational burden of the three methods have been made; a mean cycle time of 0.4 ms, 7.2 ms, and 0.1 ms for the DMP-based control, IK inverse Jacobian, and IK with swivel angle, respectively, has been estimated. As expected, IK inverse Jacobian has a higher computational burden compared the other two methods, since it is an iterative method. Conversely, it is interesting to note that the proposed DMP-based method, once trained, has a relatively low computational burden (comparable to the geometrical approach based on the swivel angle) since the DMP resolution is not computationally heavy.

The experimental results of the GCA are shown in Table 2. Mean value and standard deviation of position error, orientation error, and PhJL are reported. They were computed for GCA–sim on 238, 75, and 125 object positions (for task 1, 2, and 3, respectively) and 25 different subject anthropometries. Instead, for GCA–real they were calculated on the four subjects and 3 object positions for each task. It is interesting to note that performance achieved in the real setting are very close to the simulation results; moreover, the proposed motion planning based on DMP has a high generalization level with respect to the different object positions and subject anthropometries, since the success rate achieved for the 3 task is 100%.

**Table 2:** Experimental results obtained for GCA.

		GCA–sim	GCA–real
Task 1	Position Err [mm]	2.7 ± 0.4	3.9 ±0.5
	Orientation Err [rad]	0.14 ±0.01	0.164 ±0.008
	PhJL	0.51 ±0.03	0.64 ±0.04

Task 2-1	Position Err1 [mm]	3.2 ±0.9	4.0 ± 3.1
	Orientation Err1 [rad]	0.10 ±0.07	0.116 ±0.05
Task 2-2	Position Err2 [mm]	19 ±6	21 ±5
	Orientation Err2 [rad]	0.57 ±0.07	0.5 ±0.1
Task 2	PhJL	0.56 ±0.04	0.64 ±0.05

Task 3	Position Err [mm]	7.3 ±1.2	9.5 ±1.9
	Orientation Err [rad]	0.157 ±0.02	0.14 ±0.02
	PhJL	0.6 ±0.4	0.5 ±0.36
	Success rate [%]	100	100

## 4 Discussions

The comparative analysis (Figure 11) showed that the IK inverse Jacobian has better performance than the DMP-based control in terms of position and orientation error, but it does not guarantee physiological configuration and always the success of the operation in the whole human-robot workspace. Conversely, the IK with swivel angle reached better results than DMP-based control in terms of position and orientation error for tasks requiring the control of only one orientation parameter (e.g., tasks 1, 2-1 and 3). Instead, it increased in more complex tasks that required the control of more than one orientation parameter (task 2-2).

Nevertheless, it is worth pointing out that the position error obtained with the DMP-based control (even though higher than the traditional approaches) is fully compatible with the considered application domain which does not require very high accuracy. In fact, it is shown in the literature that accuracy of human movements during the execution of ADLs is around 1-2 cm (Merlo et al., 2013). The achieved position error is moreover well balanced by the very high success rate and the guarantee of an anthropomorphic configuration (which also entail system reliability and safety during the task fulfilment).

Furthermore, the high generalization level of the proposed approach ensures higher robustness to the environmental changes than the two other traditional methods, especially the one based on the computation of the swivel angle, which needs to be *a priori* specified. A geometrical approach for inverting kinematics (Section 2.4) has the clear advantage of a low computational burden, but it is not guaranteed that it can be easily applied on all the kinematic chains. Conversely, the proposed DMP based method offers the advantage of being applicable to any kinematic chain, thanks to the offline training, and has a good computational time (which is comparable with the IK swivel angle and significantly lower than the IK algorithm with inverse Jacobian).

## 5 Conclusion

A learning by demonstration method for planning motion of upper-limb exoskeletons was presented in this work. It is grounded on the computation of DMPs and machine learning techniques to construct the task- and patient-specific joint trajectories based on the learnt trajectories. Distinctive features, namely the DMP parameters, were firstly extracted from the motion recorded during certain activities performed by a human subject wearing the upper-limb exoskeleton. They were subsequently used, together with the recorded joint angles and Cartesian positions, to train a supervised neural network (a two layer feed-forward network). The neural network provided the more appropriate set of DMP parameters to generate the task- and patient-specific trajectories of the exoskeleton joints.

The proposed motion planning was preliminarily validated in simulation and later experimentally validated on 4 patients with LGMDs, who used the combined M-IMU/EMG interface for controlling the upper-limb exoskeleton. The validation session was aimed to (i) assess performance of the proposed motion planning system by means of quantitative indicators and compare it with traditional methods used to operate upper-limb exoskeletons, which are based on path planning and inverse kinematics (IK inverse Jacobian and IK swivel angle); (ii) investigate the generalization level of the proposed approach with respect to the variability in the experimental scenario, given for example by different anthropometry of the patients and different object positions.

The results achieved for the comparative analysis showed that the DMP-based control guarantees a 100% success rate in the task fulfillment, with an acceptable position and orientation error for the targeted application. Moreover, it also ensures that the exoskeleton always has configurations within the physiological joint limits, differently from methods based on path planning and inverse kinematics. Furthermore, the computational time required by the proposed approach is lower than the one required by the IK algorithm with inverse Jacobian and comparable with the IK with swivel angle.

Finally, the results achieved in simulation as well as in the experimental setting also showed a high generalization level of the DMP based motion planning with respect to the different object positions and subjects anthropometries. A success rate of 100% for all tasks was reported.

Future works will be addressed to extend the study to a higher number of patients and grasping and manipulation tasks, by applying the proposed motion planning approach also to the hand exoskeleton (which in this study was used to perform grasping tasks).

## Ethics Statement

The experimental protocol was approved by the local Ethical committee (Comitato Etico Università Campus Biomedico di Roma, reference number: 01/17 PAR ComEt CBM), by the Italian Ministry of Health (Registro—classif. DGDMF/I.5.i.m.2/2016/1096) and complied with the Declaration of Helsinki. All subjects gave written informed consent in accordance with the Declaration of Helsinki.

## Author Contributions

CL designed the paper, analyzed the literature, designed and developed the proposed motion planner, analyzed the experimental data and wrote the manuscript. FC organized the experimental sessions, acquired the data, analyzed the literature and contributed to the manuscript writing. ALC analyzed the literature and partly contributed to the manuscript writing. ET and SC designed and developed the IK method used as benchmark for the comparative analysis, i.e., IK based on the computation of the swivel angle, and partly contributed to the manuscript writing. JMC and FJB designed and developed the wrist-module and the hand exoskeleton. SMP recruited the patients. SS, NV, NGA contributed to the design of the experiments and discussed the results. LZ contributed to the design of the motion planner and the experiments, discussed the results, wrote the paper and supervised the study. All the authors read and approved the final version of the manuscript.

## Conflict of Interest Statement

The authors declare that the research was conducted in the absence of any commercial or financial relationships that could be construed as a potential conflict of interest.
